# Sox2 interacts with Atoh1 and Huwe1 loci to regulate Atoh1 transcription and stability during hair cell differentiation

**DOI:** 10.1371/journal.pgen.1011573

**Published:** 2025-01-30

**Authors:** Yen-Fu Cheng, Judith S. Kempfle, Hao Chiang, Kohsuke Tani, Quan Wang, Sheng-Hong Chen, Danielle Lenz, Wei-Yi Chen, Wenjin Wu, Marco Petrillo, Albert S. B. Edge

**Affiliations:** 1 Department of Otolaryngology, Harvard Medical School, Boston, Massachusetts, United States of America; 2 Eaton-Peabody Laboratory, Massachusetts Eye and Ear, Boston, Massachusetts, United States of America; 3 Speech and Hearing Bioscience and Technology, Harvard Medical School, Boston, Massachusetts, United States of America; 4 Lab for Cell Dynamics, Institute of Molecular Biology, Academia Sinica, Taipei, Taiwan; 5 National Center for Theoretical Sciences, Physics Division, Taipei, Taiwan; 6 Institute of Biochemistry and Molecular Biology, National Yang Ming Chiao Tung University, Taipei, Taiwan; 7 Harvard Stem Cell Institute, Cambridge, Massachusetts, United States of America; University of California San Francisco, UNITED STATES OF AMERICA

## Abstract

Stem cell pluripotency gene *Sox2* stimulates expression of proneural basic-helix-loop-helix transcription factor *Atoh1*. *Sox2* is necessary for the development of cochlear hair cells and binds to the *Atoh1* 3’ enhancer to stimulate *Atoh1* expression. We show here that *Sox2* deletion in late embryogenesis results in the formation of extra hair cells, in contrast to the absence of hair cell development obtained after *Sox2* knockout early in gestation. *Sox2* overexpression decreased the level of Atoh1 protein despite an increase in *Atoh1* mRNA. Sox2 upregulated E3 ubiquitin ligase, *Huwe1*, by direct binding to the *Huwe1* gene. By upregulating its cognate E3 ligase, *Sox2* disrupts the positive feedback loop through which Atoh1 protein increases the expression of *Atoh1*. We conclude that *Sox2* initiates expression, while also limiting continued activity of bHLH transcription factor, Atoh1, and this inhibition represents a new mechanism for regulating the activity of this powerful initiator of hair cell development.

## Author summary

Regeneration of hair cells, the sensory cells of the cochlea, is a potential treatment for deafness. Here, we studied the regulation of *Atoh1*, a transcription factor required for the development of hair cells in the embryo. We discovered a dual role for *Sox2* in determining the level of *Atoh1* expression in the cochlea. *Sox2* initiates the expression of *Atoh1*, while also imposing a limit on the level of Atoh1 protein through concurrent activation of the proteasome pathway. This mechanism of *Atoh1* regulation explains the variable phenotypes induced by *Sox2* deletion: a lack of hair cells when *Sox2* was deleted early in embryogenesis was attributed to the missing stimulation by *Sox2* of *Atoh1* expression, while *Sox2* deletion at later times in development resulted in an overproduction of hair cells due to decreased degradation of Atoh1 at the lowered level of E3 ubiquitin ligase *Huwe1*. This seemingly contradictory signaling enables *Sox2* to direct the fate of inner ear progenitor cells through a brief burst of *Atoh1* gene expression followed immediately by degradation of the protein.

## Introduction

*Sox2* is a core transcriptional regulator of embryonic stem cells [1,2]. It acts as a pioneer factor to allow transcription at otherwise closed chromatin [3] and binds together with partners to pro-differentiation genes, providing the cues for transitioning from progenitors to the many specialized cell types of individual organs. In the developing nervous system Sox2 binds to enhancers of proneural transcription factors to allow differentiation of neural progenitors to neurons [3–15], including transcription factors that control the differentiation of key cell types in the nervous system [9,10,15,16]. It plays a critical role in the differentiation of cochlear hair cells, the receptor cells for hearing, by stimulating expression of basic helix-loop-helix (bHLH) transcription factor, *Atoh1* [10,13], whose expression is required for development of hair cells [17]. Mammalian hair cells are subject to loss due to autoimmune disease, ototoxic medications, exposure to noise, and aging; moreover, the cells do not regenerate [18,19]. Several regulatory pathways have been found to be involved in *Atoh1* regulation [9,20–24]. Reprogramming of cochlear supporting cells to hair cells with *Atoh1* is a route to hair cell regeneration in the newborn [25–28] and adult [29–31] cochlea.

We had previously shown that destabilization of Atoh1 appeared to be critical to achieve the tight control necessary for development of the intact sensory organ. Deletion of the E3 ligase *Huwe1* which we had identified as the relevant ligase for cochlear degradation of Atoh1 [32,33] resulted in higher levels of Atoh1 protein and extra inner hair cells in the cochlea. Disruption of the *Huwe1-Atoh1* signaling pathway, by stabilizing Atoh1, induced the generation of extra hair cells from supporting cells in the developing and early postnatal cochlea.

Although *Sox2* increases the level of *Atoh1* transcript [10], we show here that, unexpectedly, it concurrently decreases the level of Atoh1 protein. To determine whether *Sox2* lowered the level of Atoh1 through a degradative pathway, we asked if inhibition of proteasomal activity would rescue Atoh1 from *Sox2*-mediated loss. Both silencing of *Huwe1*, as well as proteasome inhibition, indeed, prevented the loss of Atoh1 initiated by *Sox2*, indicating an essential role for Huwe1 in the downstream pathways activated by Sox2. Thus, we show that in addition to its role in stimulating *Atoh1*, *Sox2* initiates differentiation by Atoh1 upregulation, but then limits Atoh1 activity by co-activating its E3 ligase.

## Results

### Conditional deletion of *Sox2* produces an extra row of hair cells

We have previously shown that *Sox2* activates transcription of bHLH transcription factor, *Atoh1*, in a dose-dependent manner by binding to the Atoh1 3’ enhancer; deletion of *Sox2* prior to the differentiation of hair cells at embryonic day 13 (E13) resulted in a decrease in hair cell number by attenuating the expression of *Atoh1* [10].

**Fig 1:**
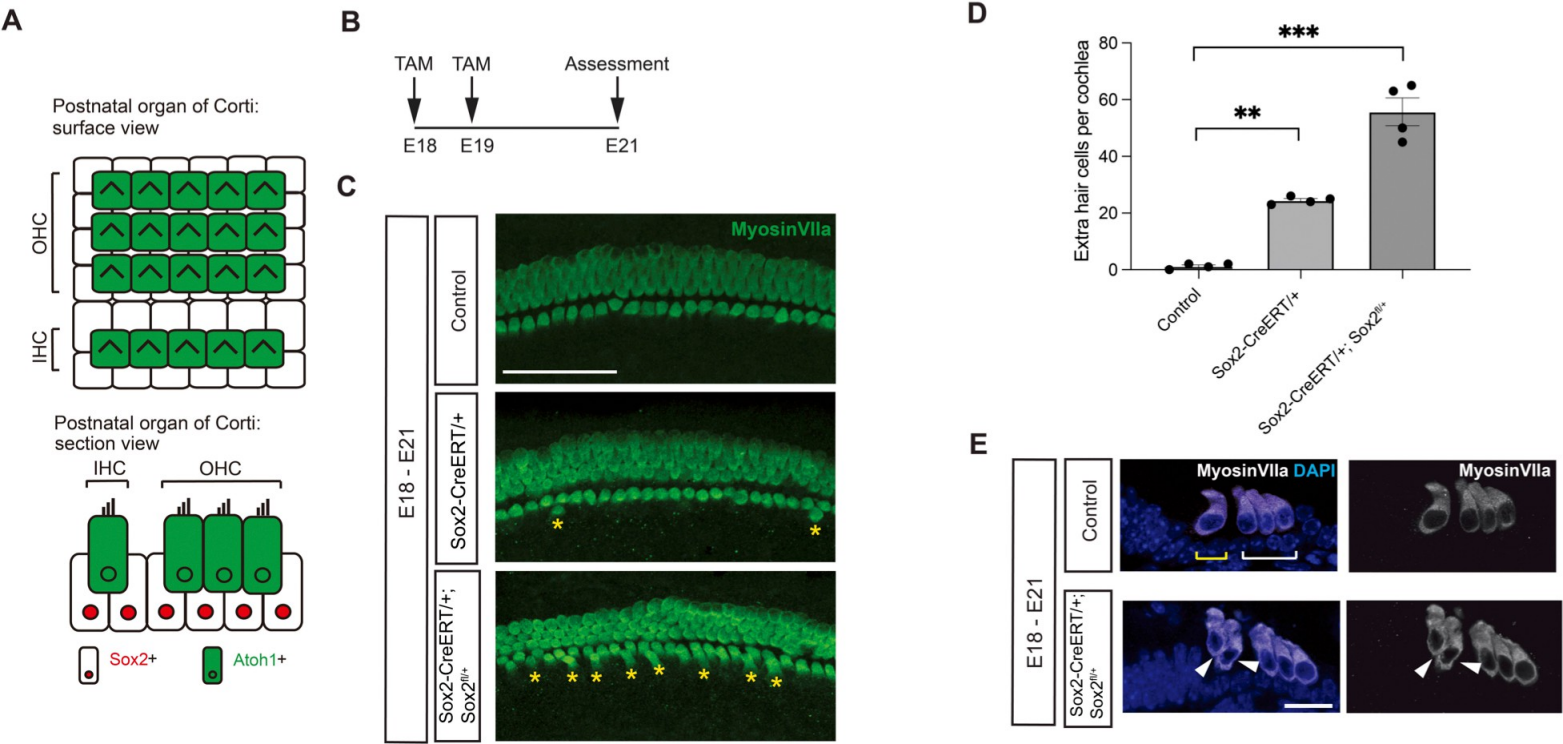
Conditional knockout of *Sox2* gives rise to extra inner hair cells. **(A)** Diagram shows the surface and section views of the postnatal cochlea. The sensory epithelium is composed of 3 rows of outer hair cells and 1 row of inner hair cells (green cells expressing *Atoh1*) surrounded by supporting cells (white cells with red nucleus expressing Sox2). **(B)** Timeline of tamoxifen (TAM) injection and assessment of the effect of Sox2 knockout. **(C)** Extra hair cells were apparent (yellow asterisks) after *Sox2* knockout at E18 in *Sox2-CreERT/+*;*Sox2*^*fl/+*^ embryos (also referred to as *Sox2 knockouts)* and *Sox2-CreERT/+* embryos (also referred to as *Sox2 hypomorphs)*. The additional hair cells were apparent in a whole mount of the cochlea in a *Sox2* knockout, whereas a *Cre*-negative littermate control had only a single row of inner hair cells and a heterozygous knockout had a small number of extra inner hair cells. Myosin VIIa labels hair cells. Scale bar = 100 μm. **(D)** Quantification of extra hair cells. Extra hair cells from control and *Sox2* conditional knockout cochlea from (B) are shown (n = 4 for each group). Error bars indicate SEM. **p < 0.01, ***p < 0.001. **(E)** Examination of the cochlea at E21 after *Sox2* deletion at E18 showed an extra row of hair cells (arrowheads). Hair cells expressed myosin VIIa (white). Nuclei were labeled with DAPI in blue. A littermate control (*Cre*-negative) with a single allele of *Sox2* had normal hair cells. White bracket indicates outer hair cells and yellow bracket indicates inner hair cells. Scale bar = 25 μm.

The sensory epithelium in the late embryonic and postnatal cochlea consists of 3 rows of outer hair cells and 1 row of inner hair cells surrounded by supporting cells (Fig 1A). In contrast to knockout of *Sox2* before hair cell differentiation, *Sox2* knockout at a time point when hair cells had already undergone terminal differentiation (E18) increased the number of hair cells (Fig 1B–1D). The effect of *Sox2* deletion at E18 on the number of hair cells in the cochlea was dose-dependent: the number of hair cells seen after homozygous deletion of *Sox2* was greater than after heterozygous deletion or a wild-type cochlea (Fig 1C; 55.75 vs 24. 5 vs 1.25 extra hair cells per cochlea, p < 0.001). The new hair cells in the conditional knockout were adjacent to the inner hair cells (Fig 1B–1D). The addition of hair cells after *Sox2* conditional knockout late in embryonic development suggested that deletion had a changed role compared to early embryonic time points where a loss of hair cells has been observed [10].

### *Sox2* concomitantly regulates *Huwe1* and *Atoh1*

*Atoh1* is expressed in the developing organ of Corti [17], where it is required for the differentiation of hair cells, the receptor cells for the sense of hearing. Atoh1 participates in a positive feedback loop in which the protein binds to an E-box in the enhancer 3’ of the coding region and thereby upregulates Atoh1 transcription [34]. We have found that Atoh1 is turned over rapidly and that this turnover is stimulated by *Huwe1* [32]. Huwe1 has been identified as the ubiquitin E3 ligase for Atoh1 in the cerebellum and the cochlea [32,33], targeting Atoh1 for destruction by the proteasome.

**Fig 2:**
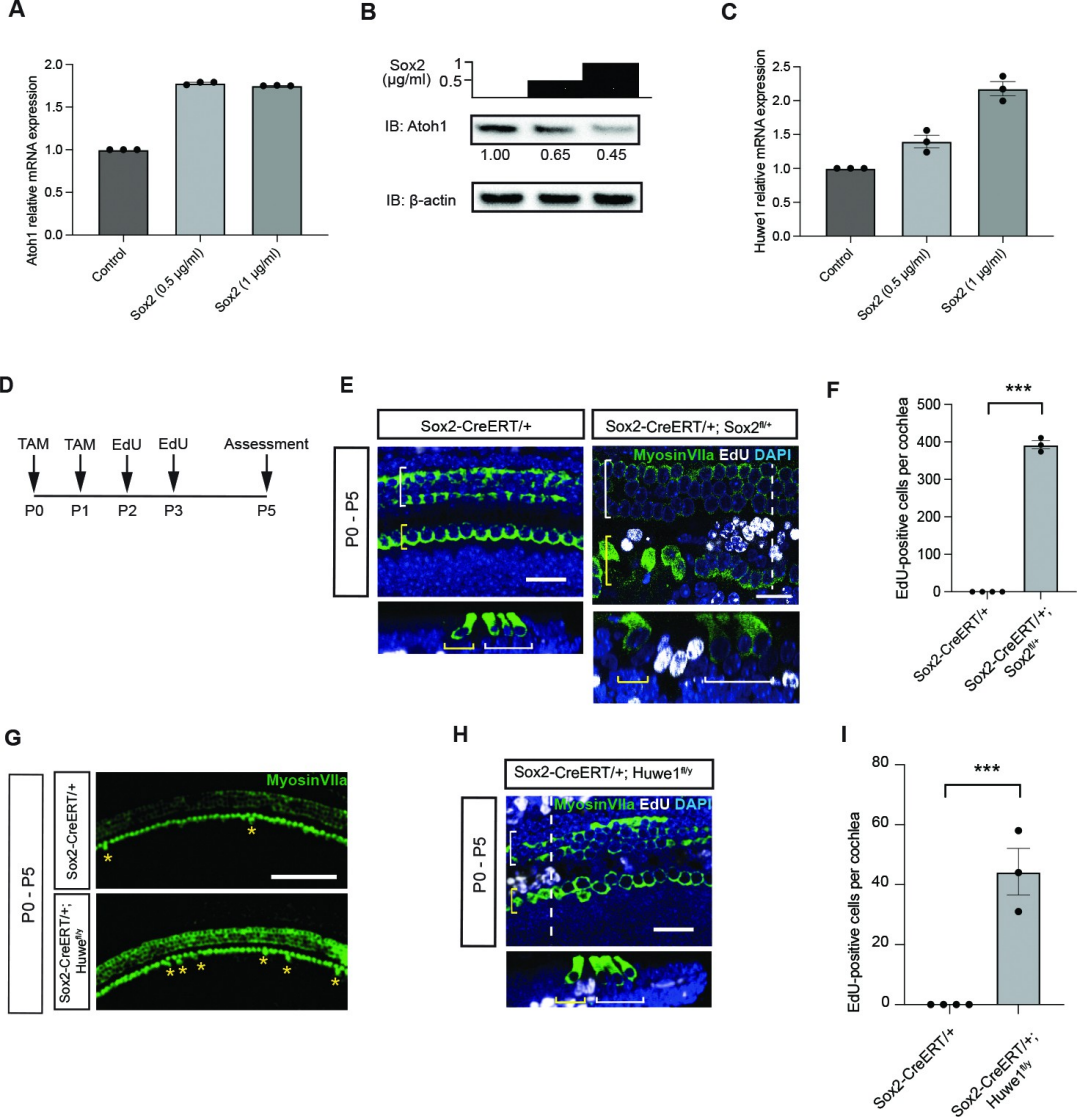
*Sox2* stimulates degradation of Atoh1 through ubiquitin E3 ligase, *Huwe1*, and knockout of *Sox2* or *Huwe1* results in supporting cell division. **(A)** qRT-PCR analysis of *Atoh1* after *Sox2* overexpression in cochlear cells. OC-1 cells were transfected with the indicated concentration of *Sox2*. Lysates were subjected to qRT-PCR. Error bars indicate SEM (n = 3 experiments). **(B)** Downregulation of Atoh1 protein by *Sox2* overexpression. OC-1 cells were transfected with the indicated concentrations of *Sox2*. Lysates were processed for Western blotting with Atoh1 or β-actin (loading control) antibodies. Atoh1 levels (determined by densitometry) are shown below each lane. **(C)** qRT-PCR analysis of *Huwe1* in OC-1 cells. The samples from (**B**) were analyzed by qRT-PCR. (n = 3 experiments). **(D)** Timeline of tamoxifen and EdU induction. Tamoxifen (TAM) was injected for two consecutive days, followed by EdU. **(E)** EdU incorporation in *Sox2*-deleted cochlea at P5. A *Sox2-CreERT/+;Sox2*^*fl/+*^ cochlea examined at P5 after deletion of *Sox2* by administration of tamoxifen at P0 and EdU at P2 had numerous proliferated supporting cells. White bracket indicates outer hair cells and yellow bracket indicates inner hair cells. An orthogonal scan was performed at the position indicated by the white dotted line. Scale bars = 25 μm. **(F)** Quantitative analysis of EdU-positive cells. Error bars indicate SEM (*Sox2*^+/-^, n = 4 experiments; *Sox2-CreERT/+;Sox2*^*fl/+*^, n = 3 experiments; ***p < 0.001). **(G)** Extra hair cells were apparent (yellow asterisks) after *Huwe1* knockout in Sox2-positive supporting cells at P0, examined at P5. The additional hair cells were apparent in a whole mount of the cochlea in a *Huwe1* knockout. Myosin VIIa labels hair cells. Scale bar = 100 μm. **(H)** EdU staining of *Sox2-CreERT/+;Huwe1*^*fl/y*^ at P5. Tamoxifen was induced at two consecutive days, P0 and P1 followed by EdU at P2 and P3. White bracket indicates outer hair cells and yellow bracket indicates inner hair cells. An orthogonal scan was performed at the position indicated by the white dotted line. EdU positive cells could be observed in *Sox2-CreERT/+;Huwe1*^*fl/y*^ cochlea. Scale bar = 25μm. **(I)** Quantitative analysis of EdU positive cells in the pillar cell area. Error bars indicate SEM (n = 4 experiments; ***p < 0.001).

*Atoh1* expression responds to the level of *Sox2* [10]. To resolve the difference in the role of Sox2 at different times of development, we explored the role of Sox2 in the control of Atoh1 expression. Assessment of the biochemical interactions and mechanism of *Sox2*-mediated downregulation of Atoh1 protein were performed in OC-1 cells. Consistent with previous results [9–11,13,15,16], overexpression of *Sox2* led to a dose-dependent increase in *Atoh1* (Fig 2A). However, examination of the protein by Western blotting revealed a post-translational downregulation of Atoh1 by *Sox2* (Fig 2B). Both the upregulation of mRNA and the decrease in protein with *Sox2* overexpression were dependent on the level of *Sox2* (Fig 2B). Thus, we found an inverse relationship, in which *Sox2* caused an increase in mRNA but a decrease in Atoh1 protein over the course of 24 hours of *Sox2* overexpression. In addition, we observed an increase in *Huwe1* with Sox2 expression in these cells (Fig 2C). We hypothesized that the paradoxical dose-dependent decrease in Atoh1 protein could be due to the Sox2-dependent increase in Huwe1.

*Sox2* levels increased as did *Huwe1*, in response to doxycycline in a tetracycline-inducible transgenic embryonic stem-cell line in which we could overexpress *Sox2* [35]. *Atoh1* was also increased, but the increase was not monotonic and decreased at higher levels of *Sox2* when Huwe1 continued to increase (S1 Fig). This suggested that Sox2 was acting on pathways that decreased the level of Atoh1 protein while concurrently stimulating transcription of the bHLH transcription factor.

To assess whether the new hair cells resulting from *Sox2* knockout may have originated from supporting cell division, we injected EdU at P2, after tamoxifen administration to knock out *Sox2* at P0 (Fig 2D) and assessed cochlear immunostaining (Fig 2E). The organ of Corti after *Sox2* knockout in *Sox2ERT;Sox2*^*fl/+*^ animals that received tamoxifen from the lactating mother at P0 contained EdU-labeled cells in the P5 cochlea (Fig 2F). Extra supporting cells arose from supporting cells lateral to the inner hair cells, in the region of the pillar cells (Fig 2E and 2F). This showed that the phenotype was due to supporting cell division, and suggested that after the deletion of *Sox2*, new hair cells could differentiate from supporting cells directly or after proliferation.

*Huwe1* knockout mice treated with tamoxifen at P0 also showed a supporting cell division phenotype. Extra hair cells were seen in the *Huwe1* knockout at P5 (Fig 2G and 2H) similar to the results of our previous work showing that *Huwe1* knockout affected hair cell generation in the cochlea [32]. While supporting cells in the *Huwe1* knockout cochlea underwent cell division in response to the gene knockout, similar to the *Sox2* knockout (Fig 2I), the extent of cell division was 10X lower, which was attributable to the broader downstream targets expected for *Sox2* compared to *Huwe1* [32].

### *Sox2* is upstream of *Huwe1* and *Atoh1* and *Sox2* deletion induces cochlear supporting cell conversion to hair cells

**Fig 3:**
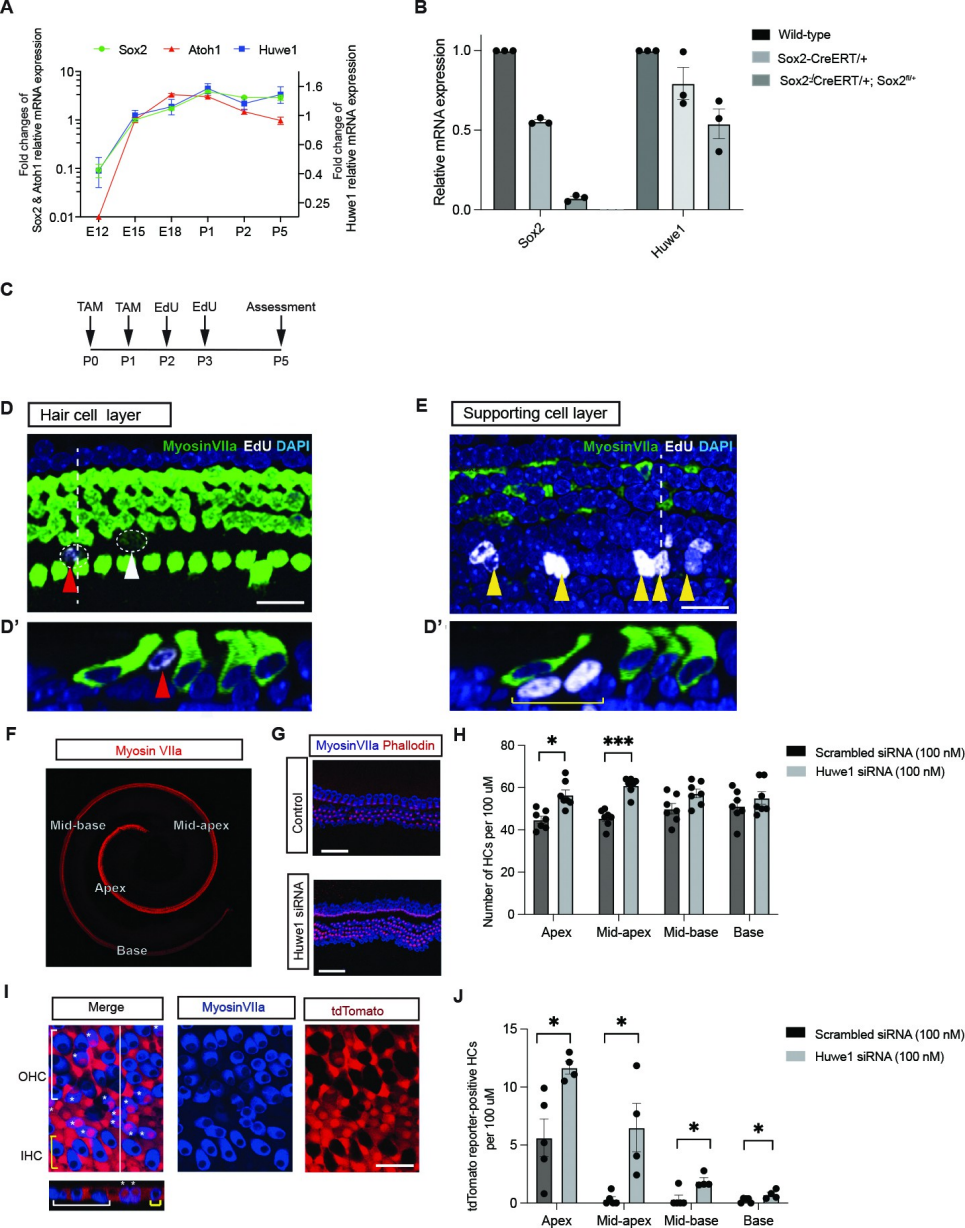
Sox2 is upstream of *Atoh1* and *Huwe1* in embryonic development and *Huwe1* knockdown results in hair cell generation from dividing supporting cells. **(A)** qRT-PCR showed that *Huwe1* and *Sox2* were expressed in the cochlea at E12. *Atoh1* was not detected at that time point but was detected at E15. *Sox2* was expressed before *Atoh1* and was maintained throughout the downregulation of *Atoh1*. *Huwe1* was expressed prior to *Atoh1*, and expression was also maintained. Relative expression levels were compared with E15 and normalized to *Gapdh*. Error bars indicate SEM (n = 3 experiments). **(B)** *Huwe1* expression was downregulated after conditional knockout of *Sox2* in the embryonic cochlea. Female *Sox2*^*fl/+*^mice were mated with *Sox2-CreERT/+* males and received tamoxifen at E17.5 and E18.5. Embryonic cochlea was analyzed by qRT-PCR at E21. Error bars indicate SEM (n = 3 experiments). **(C)** Timeline of tamoxifen and EdU induction. **(D)** The EdU-labeled cells were seen at P5 in the hair cell layer after *Huwe1* deletion in a *Sox2-CreERT/+;Huwe1*^*fl/y*^ cochlea induced by tamoxifen at P0. An orthogonal scan was performed **(D’)** at the position indicated by the white dashed line. Scale bar = 25 μm. **(E)** Optical section at the supporting cell level shows EdU-positive supporting cells (yellow arrowheads) after administration of EdU at P2 with tamoxifen at P0. An orthogonal scan was performed **(E’)** at the white dashed line. The yellow bracket indicates newly generated supporting cells. Scale bar = 25 μm. **(F)** Neonatal organ of Corti is divided into 4 regions. **(G)** Effect of *Huwe1* knockdown on the organ of Corti. Organs of Corti treated with *Huwe1* siRNA (100 nM) for 72 hours have increased numbers of hair cells, which are marked by myosin VIIa. The supernumerary hair cells are positive for phalloidin, a hair bundle marker. The scale bar is 100 μm. **(H)** Quantification of hair cell counts in the apex, mid-apex, mid-base, and base (mean ± SEM per 100 mm; *p < 0.05, ***p<0.001, n = 7 for both groups). **(I)** Double-labeled cells positive for the tdTomato reporter (red) and myosin VIIa (blue) were found in the pillar area in the mid-apex region of cochlear tissue from neonatal mice carrying the *Sox2-CreERT* as well as the tdTomato reporter 3 days after treatment with *Huwe1* siRNA (100 nM). Td-Tomato reporter in myosin VIIa-positive cells indicates hair cells that arose from supporting cells. Scale bar = 25 μm. **(J)** Effect of *Huwe1* knockdown was significant. Quantification of the tdTomato reporter-positive hair cells in scrambled and *Huwe1*-siRNA treated explants showed significantly more reporter-labeled hair cells after *Huwe1* siRNA treatment (mean ± SEM per 100 mm; *p < 0.05, n = 4 for both groups).

*Sox2* and *Huwe1* were both detected in the developing mouse cochlea at E12, the earliest time tested (Fig 3A) and both increased to a maximum at E18 and were then maintained to P5, the last time tested, whereas *Atoh1* was first detected at E15 and began to decrease at P1. The first expression of *Sox2* is consistent with a role upstream of *Atoh1*, and the expression of *Huwe1* is consistent with a role in the degradation of Atoh1. *Sox2* was also seen in the early prosensory epithelium and persisted in supporting cells and hair cells through P0, followed by its restriction to supporting cells in the postnatal animal [10]. A similar effect of Huwe1 knockdown with siRNA was observed for Atoh1. We therefore hypothesized that the Huwe1-sensitive degradation of Atoh1 seen after *Sox2* overexpression was due to *Sox2*-activated *Huwe1* expression.

*Sox2* knockout in the organ of Corti by administration of tamoxifen to a *Sox2-CreERT/+;Sox2*^*fl/+*^ mouse, which deletes *Sox2* in supporting cells of the cochlea, decreased *Huwe1* expression (Fig 3B). Our observation that *Sox2* overexpression upregulated *Huwe1*, combined with the finding that *Sox2* deletion in the late embryo decreased the expression of *Huwe1*, suggested that *Sox2* was acting on Atoh1 through *Huwe1*, and the decreased *Huwe1* expression in the absence of *Sox2* could also explain the phenotype of extra hair cells seen after *Sox2* knockout (Fig 1). We therefore further explored the idea that *Sox2* controlled the proteasomal degradation of Atoh1.

The organ of Corti after *Huwe1* knockout in *Sox2-CreERT/+;Huwe1*^*fl/y*^ animals that received tamoxifen from the lactating mother at P0 (Fig 3C) contained EdU-labeled cells (Fig 3D and 3E). The EdU-labeled cells were found in the supporting cell layer (Fig 3D), and, again, these cells appeared to be derived from supporting cells on the lateral aspect of the inner hair cells (Fig 3E and 3E’). This suggested that, like deletion of *Sox2*, deletion of *Huwe1* resulted in new hair cells via supporting cell division.

We then used an explant model in which the organ was removed at P0 followed by *Huwe1* knockdown with siRNA to show that extra hair cells were formed at the apex and mid-apical regions of the cochlea (Fig 3F–3H). We further showed the extra hair cells arose from supporting cells that differentiated to hair cells in response to gene knockdown (Fig 3I–3J; tdTomato-myosin VIIa, double-labeled cells) and that the new hair cells were in the region between the inner and outer hair cells (Fig 3I), similar to the extra hair cells from the *Sox2* knockout (Fig 1).

### *Sox2* upregulates *Huwe1* by direct interaction with its promoter and acts through *Huwe1* to downregulate Atoh1 in cochlear organoids

We next asked whether *Sox2* altered *Huwe1* expression by interacting directly with the *Huwe1* gene, the mechanism it uses for regulation of numerous downstream genes, including *Atoh1* [10]. We generated inner ear organoids from Lgr5-expressing supporting cells of the cochlea utilizing our previously established procedure [36]. The organoids comprise Lgr5-expressing cochlear progenitor cells capable of differentiating in high yield to hair cells and allow downstream analysis for transcription factor activities. In these experiments we used cochlear sensory epithelium dissected from P3 pups to generate organoids [36]. We assessed Sox2 binding to chromatin after differentiation of the organoids for 2 days, when *Atoh1* is actively expressed.

**Fig 4:**
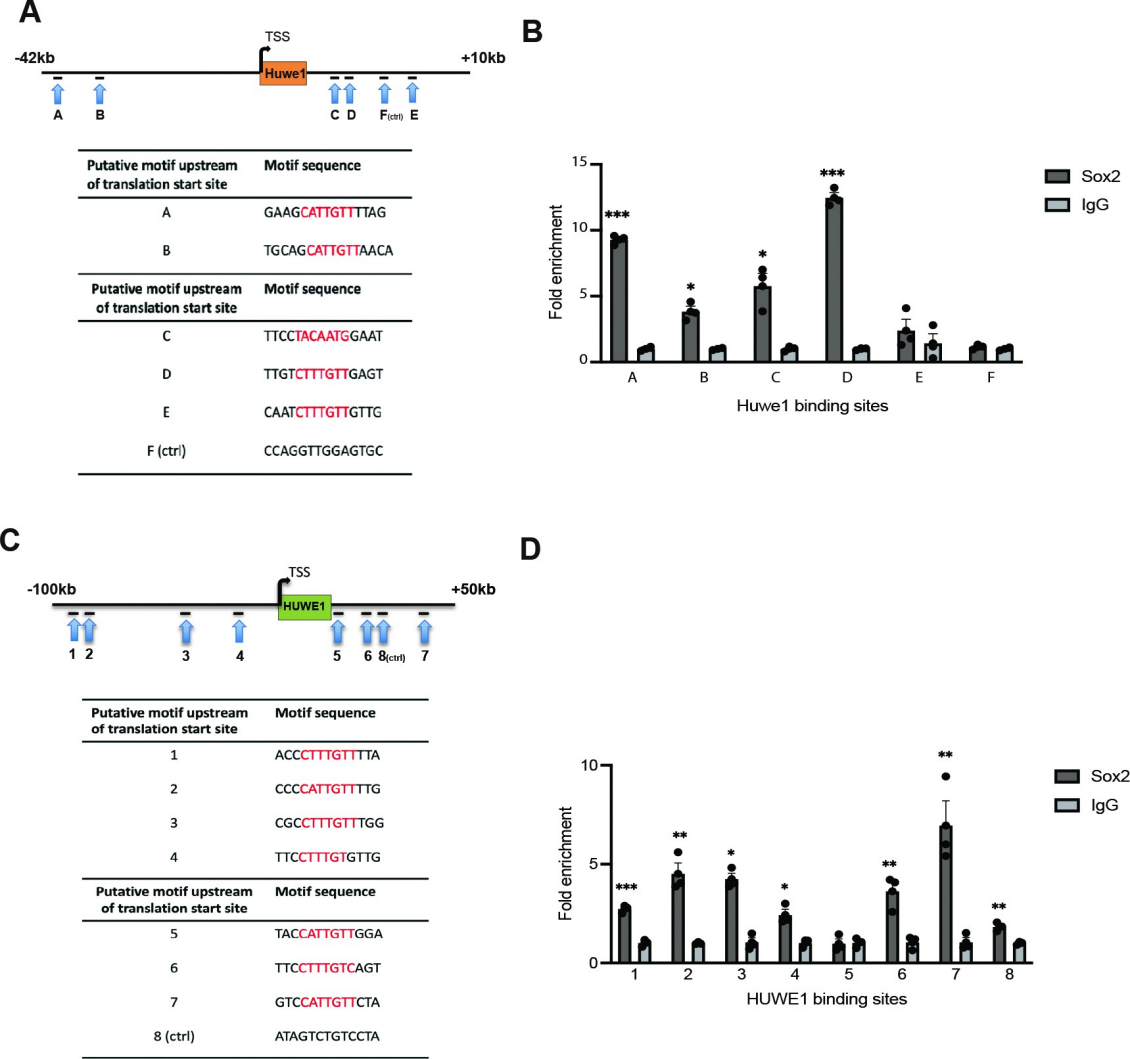
Sox2 direct interaction with mouse *Huwe1* and human *Huwe1* genes. **(A)** Schematic diagram shows four putative Sox2-binding motifs (1–4) in the region 100-kb upstream of the translation start site, and three putative Sox2 binding motifs (5–7) in the region 50-kb downstream of the stop codon of mouse *Huwe1*. A random motif downstream of the translation stop codon of mouse *Huwe1* (8) was the control (ctrl). **(B)** Chromatin immunoprecipitation for Sox2 binding sites at the mouse *Huwe1* locus in cochlear organoids which were proliferated for 10 days and differentiated for 2 days. The fold enrichment for each site after immunoprecipitation with an antibody to Sox2 compared to normal goat IgG is shown. Error bars indicate SEM (n = 3 experiments; *p<0.05, **p<0.01, ***p<0.005 calculated using T test). **(C)** Schematic diagram shows two putative Sox2-binding motifs (A, B) in the region 42-kb upstream of the translation start site, and three putative Sox2 binding motifs (C-E) in the region 10-kb downstream of the stop codon of human *Huwe1*. A random motif downstream of the translation stop codon of human *Huwe1* (F) was the control (ctrl). **(D)** Chromatin immunoprecipitation for Sox2 (HA) binding sites at the human *Huwe1* locus after overexpression of *Sox2-HA* in HEK 293T cells. The fold enrichment for each site after immunoprecipitation with an antibody to HA (Sox2) compared to mouse IgG is shown. Error bars indicate SEM (n = 3 experiments; *p<0.05, **p<0.01, ***p<0.005 calculated using T test).

We analyzed the sequence 100 kb upstream of the translation start site and 50 kb downstream of the stop codon of the mouse *Huwe1* gene for Sox2 binding motifs and identified 4 sites upstream, and 3 sites downstream, of the coding sequence (Fig 4A). We performed chromatin immunoprecipitation (ChIP) with Sox2 antibody or IgG followed by qPCR with primers for the Sox2 binding sites and a random sequence (S1 Table). We detected enrichment of the Sox2 binding at seven Sox2 binding motifs. Compared to the random site, the other Sox2 binding site, especially site 7, showed largest enrichment, suggesting that Sox2 directly interacted with the *Huwe1* gene in LCPs (Fig 4B).

To explore whether Sox2 altered *Huwe1* expression by interacting directly with the *Huwe1* gene, we assessed the Sox2 binding in HEK 293T cells in which we efficiently overexpressed Sox2-HA plasmid. We analyzed the sequence 42kb upstream of the translation start site and 10 kb downstream of the stop codon of the human *Huwe1* gene for Sox2 binding motifs and identified 2 sites upstream, and 3 sites downstream, of the coding sequence (Fig 4C). We performed chromatin immunoprecipitation (ChIP) with HA antibody or IgG followed by qPCR with primers for the Sox2 binding sites and a random sequence, 6.2kb downstream of the *Huwe1* gene (S2 Table). We detected significant enrichment of the Sox2 binding at all 5 of the Sox2 binding motifs compared to the random site, and the Sox2 binding site D showed the highest enrichment, suggesting that Sox2 directly interacted with the *Huwe1* gene in HEK 293T cells (Fig 4C). *Huwe1* mRNA was upregulated by *Sox2* overexpression in HEK 293T cells, further demonstrating induction of *Huwe1* transcription by *Sox2* (Fig 4D).

### *Huwe1* is essential for *Sox2*-mediated decrease in Atoh1 protein

**Fig 5:**
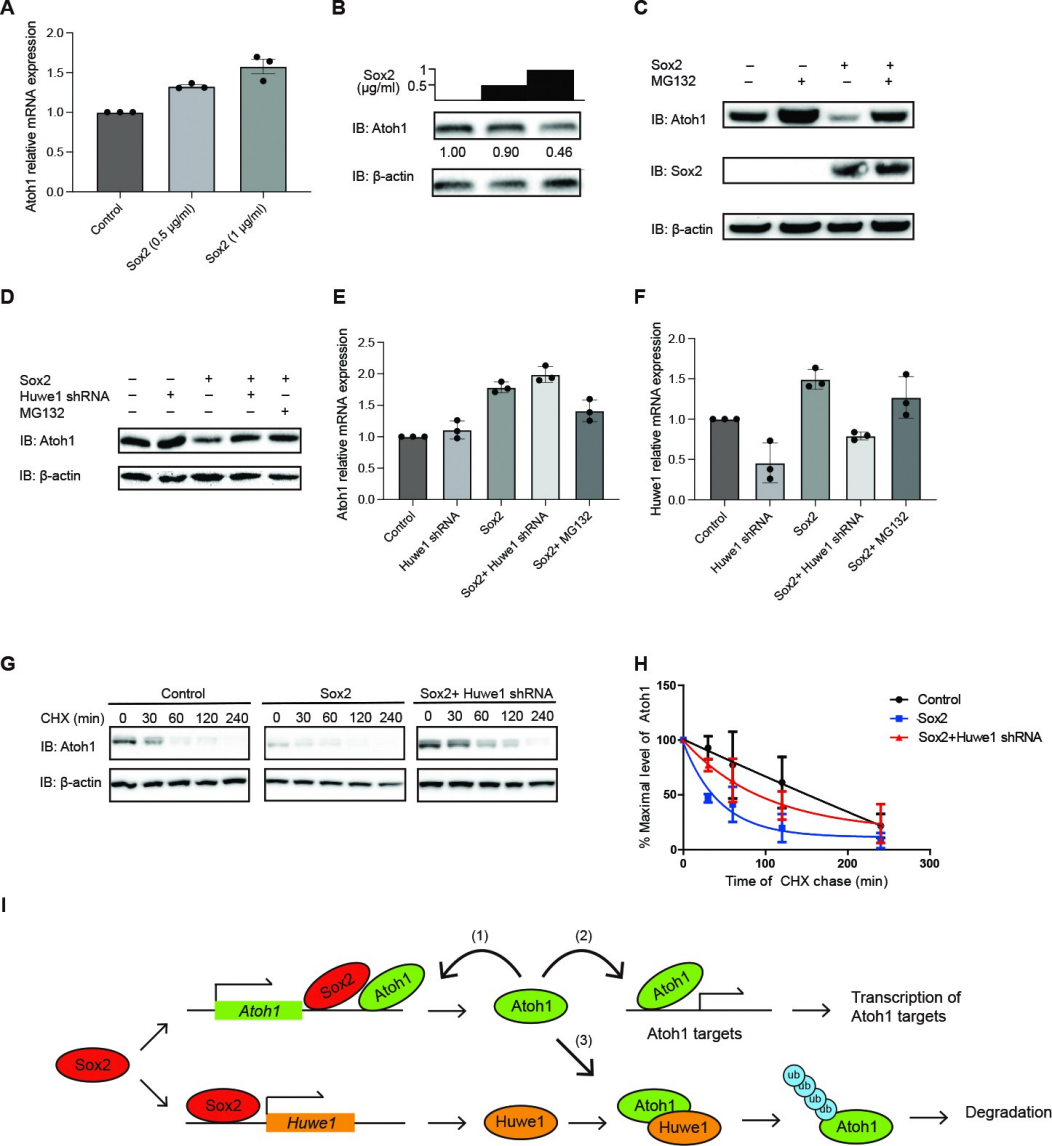
*Huwe1* is required for Sox2 stimulated degradation of Atoh1. **(A)** *Atoh1* mRNA upregulation by Sox2. HEK-293T cells were transfected with the indicated concentrations of *Sox2* followed by qRT-PCR. Error bars indicate SEM (n = 3 experiments; **p < 0.01, ***p < 0.001). **(B)** Lysates from (**A**) were processed for Western blotting with FLAG antibody (Atoh1), or β-actin antibody (loading control) and quantified by densitometry (Atoh1 levels are shown below the lanes). **(C)** Downregulation of Atoh1 by *Sox2* overexpression is rescued by proteasome inhibition. HEK 293T cells were transfected with *HA-Sox2* and/or treated with proteasome inhibitor, MG132 (10 μM), for 6 hours. After 24 hours of transfection, lysates were processed for Western blotting with FLAG antibody, Sox2 antibody, or β-actin antibody (loading control). **(D)** *Huwe1* knockdown prevented Atoh1 degradation like proteasome inhibitor, MG132. Both the baseline and the *Sox2*-induced decrease in Atoh1 in HEK 293T cells were prevented by *Huwe1* shRNA and MG132. Atoh1 levels (determined by densitometry) are shown below each lane. Lysates were processed for Western blotting with FLAG (Atoh1), Sox2, or β-actin (loading control) antibodies. **(E), (F)** qRT-PCR of *Atoh1* and *Huwe1*. *Sox2* overexpression in HEK 293T cells upregulated both *Atoh1* and *Huwe1*, while *Huwe1* shRNA and MG132 did not affect *Atoh1* mRNA. Error bars indicate SEM (n = 3 experiments). (**G**), (**H**) The decreased Atoh1 protein half-life upon *Sox2* overexpression could be rescued by *Huwe1* knockdown in a cycloheximide chase (CHX) assay in HEK 293T cells transiently transfected with *FLAG-HA-Atoh1*, *HA-Sox2*, and *Huwe1* shRNA. Error bars indicate SEM (n = 3 experiments). **(I)** *Sox2* induces *Atoh1* transcription by binding to the *Atoh1* 3’ enhancer. The Atoh1 protein binds to its 3’ enhancer to upregulate transcription in a positive feedback loop (1) and activates downstream genes (2). *Sox2* also activates transcription of E3 ligase *Huwe1*, which binds Atoh1 (3) and initiates proteasomal degradation by ubiquitylation. Degradation of Atoh1 breaks the positive feedback loop through which Atoh1 upregulates *Atoh1* transcription.

The reversal of the dose-dependent decrease in Atoh1 protein after overexpression of *Sox2* in HEK 293T cells by treatment with proteasome inhibitor, MG132 (Fig 5A–5C) indicated that the degradation of Atoh1 after *Sox2* overexpression was due to *Sox2*-activated proteasomal degradation of the protein. The decrease in Atoh1 was also reversed by shRNA targeting *Huwe1* (Fig 5D). The increase in *Atoh1* mRNA after *Sox2* overexpression was not affected by *Huwe1* shRNA or MG132 (Fig 5E); expression of *Huwe1* was only decreased by *Huwe1* shRNA as expected. Therefore, the decrease in Atoh1 caused by Sox2 was not an effect on transcription but was a posttranslational effect (Fig 5F) that could be blocked by disruption of the Huwe1-ubiquitin-proteasome pathway.

### *Sox2* lowers Atoh1 protein by an effect on Huwe1-mediated proteasomal degradation

To obtain further evidence for a role of *Sox2* in the Huwe1-mediated degradation of Atoh1, we performed cycloheximide assays to follow the fate of previously synthesized Atoh1 protein in the absence of new protein synthesis. *Sox2* overexpression in HEK 293T cells treated with cycloheximide resulted in the degradation of Atoh1 at a higher rate than in the absence of *Sox2* co-transfection (Fig 5G and 5H). The increased rate of degradation was rescued by shRNA to *Huwe1* indicating that *Sox2* influenced the rate of Atoh1 degradation by a direct effect on *Huwe1* expression. Thus, we concluded that *Sox2* regulated degradation of Atoh1 by activation of *Huwe1* (Fig 5I).

### *Sox2* binding to ubiquitin E3 ligase and transcription factor genes

To ask whether Sox2 could affect interactions between ubiquitin E3 ligases and transcription factors in other progenitor cells, we assessed databases for Sox2 binding to chromatin at both E3 ubiquitin ligase and transcription factor loci in glioblastoma cells, neural progenitor cells and embryonic stem cells (S4 Table). Sox2 binding sites in these databases included 317 ubiquitin E3 ligase genes, as well as over one thousand transcription factor genes [11,37,38]. Sox2 binding sites were localized to distal enhancer regions in these cells [11], and were consistent with a role for Sox2 in maintaining pluripotency as well as specification of neural identity as described here for the differentiation of sensory precursor cells to hair cells. Further probing of ubiquitin E3 ligases for transcription factors using UbiBrowser [39] identified 180 E3 ligases (including Huwe1) interacting with Sox2 that were predicted to serve as E3 ligases for 237 proteins (including Atoh1) (S5 Table). While insufficient to identify specific transcription factor-E3 ligase cognate interactions, the bioinformatic pairing of Sox2 binding proteins to E3 ligases suggested that this function of Sox2 may be relevant across multiple transcription factor genes.

## Discussion

*Sox2* hypomorphs and knockouts lack hair cells [40], indicating a role for *Sox2* in cochlear development. We show here that deletion of *Sox2* late in gestation, when *Atoh1* was already expressed and hair cells had undergone terminal differentiation (E18), resulted in the formation of extra hair cells. This was the same phenotype as *Huwe1* knockout [32] and was similar to the phenotype of *Atoh1* overexpression [41].

Our previous data showed that *Sox2* stimulated expression of basic-helix-loop-helix transcription factor *Atoh1* by binding to the *Atoh1* 3’ enhancer [10]. In experiments performed here, a discrepancy between increased *Atoh1* mRNA and decreased Atoh1 protein after overexpressing *Sox2* was resolved by our observation that *Sox2* upregulates E3 ubiquitin ligase, *Huwe1*, the E3 ligase that ubiquitylates Atoh1 [32,33], by direct binding to the *Huwe1* gene. The destabilization of Atoh1 by *Huwe1* in response to *Sox2* appeared to be critical for normal hair cell development. Since *Atoh1* expression is regulated by Atoh1 protein binding to the 3’ enhancer in a positive feedback loop [34], *Huwe1*-mediated destabilization of Atoh1 would disrupt the feedback loop by depleting Atoh1 protein. Consistent with disruption of the positive feedback loop, *Atoh1* expression decreases to near undetectable levels shortly after *Atoh1*-mediated differentiation [21,26].

This incoherent feedforward regulation of *Sox2* permits the maintenance of elevated steady state levels of *Atoh1*. *Sox2* is a reprogramming factor for iPS cells [42] as well as for directly induced neurons [43–45]. Activity of *Sox2* in neural differentiation is mediated by lncRNA, RMST [14], which is controlled by REST, a repressor of proneural gene expression. Sox2 is also a pioneer factor that can bind to compacted chromatin in nucleosomes [46]. It can act to decrease methylation at silenced chromatin sites and may do this in its role as a proneural pro-differentiation transcription factor [47]. The pioneer activity of *Sox2* may be important in attempts to stimulate regeneration in adult tissue where the developmental loci may be closed [48]. Low levels of proneural transcription factors, which are usually kept epigenetically bivalent to be poised for activation [49], may be neutralized by the proteasome. Hair cells extinguish *Atoh1* expression shortly after differentiation and this is followed by epigenetic silencing of the gene [50]. *Sox2* is an HMG domain transcription factor with an important role in the maintenance of stem cell pluripotency [1,42], acting as a core transcriptional regulatory factor by binding to DNA of transcription factor genes [1,2]. *Sox2* has a role in pluripotency by damping down the expression of developmental transcription factors together with pluripotency factors, Oct4 and Nanog [1,2].

However, *Sox2* also activates pro-differentiation genes and can open bivalent genes to allow their transcription. Despite the loss of *Sox2* expression prior to maturation of the chick neural tube [8] and neural differentiation [5,8,51], other studies have shown that *Sox2* activates genes required for differentiation [3,4,9–11,14,52]; for example, a defect in maturation of neurons was seen in *Sox2* hypomorphs [6]. *Sox2* plays a role in the differentiation of pluripotent progenitor cells to tissue-specific cells predominantly in the nervous system where it stimulates the expression of proneural transcription factors by binding to chromatin at specific sites in the regulatory regions of these genes.

The inhibitory effect of *Sox2* on neural differentiation has previously been explained by a variety of molecular pathways, such as the direct inhibition of proneural transcription factor expression [8,13], the upregulation of inhibitory transcription factors [5], and feedback regulation to prevent expression [7,8]. In the cochlea [7] *Sox2* was suggested to be antagonistic to *Atoh1* expression, as its overexpression blocked hair cell fate. The antagonism of *Sox2* to hair cell differentiation has been attributed to effects on Notch signaling and had focused on the stimulation of Notch downstream effectors of the *Hes/Hey* family [13].

Our data, however, suggest that destabilization of proneural transcription factors is the key mechanism for these observations, and the effect of *Sox2* on hair cell differentiation is due to destabilization of Atoh1. The resulting co-regulation of transcription factor and E3 ligase could limit the activity of pro-differentiation transcription factors after initiating cell fate through a short burst of expression. The variable results obtained in experiments on *Sox2* overexpression in neurogenesis [43–45] are likely related to the dual effects of *Sox2*, by regulating expression and protein half-life of these powerful cell fate-determining transcription factors. Thus, proteasomal degradation is needed to tune the effects of Atoh1 by regulating *Huwe1* expression when *Sox2* is expressed. Thus, the posttranscriptional regulation of Atoh1 is orchestrated by Sox2 and Huwe1 in concert with casein kinase 1, the kinase that phosphorylates Atoh1 [32], thereby tagging it for degradation. This view sheds light on the observations that *Sox2* is required for the differentiation of a variety of cell types [3,4,9–11,14,47,52], including neurons [6] and sensory cells [10].

**Fig 6:**
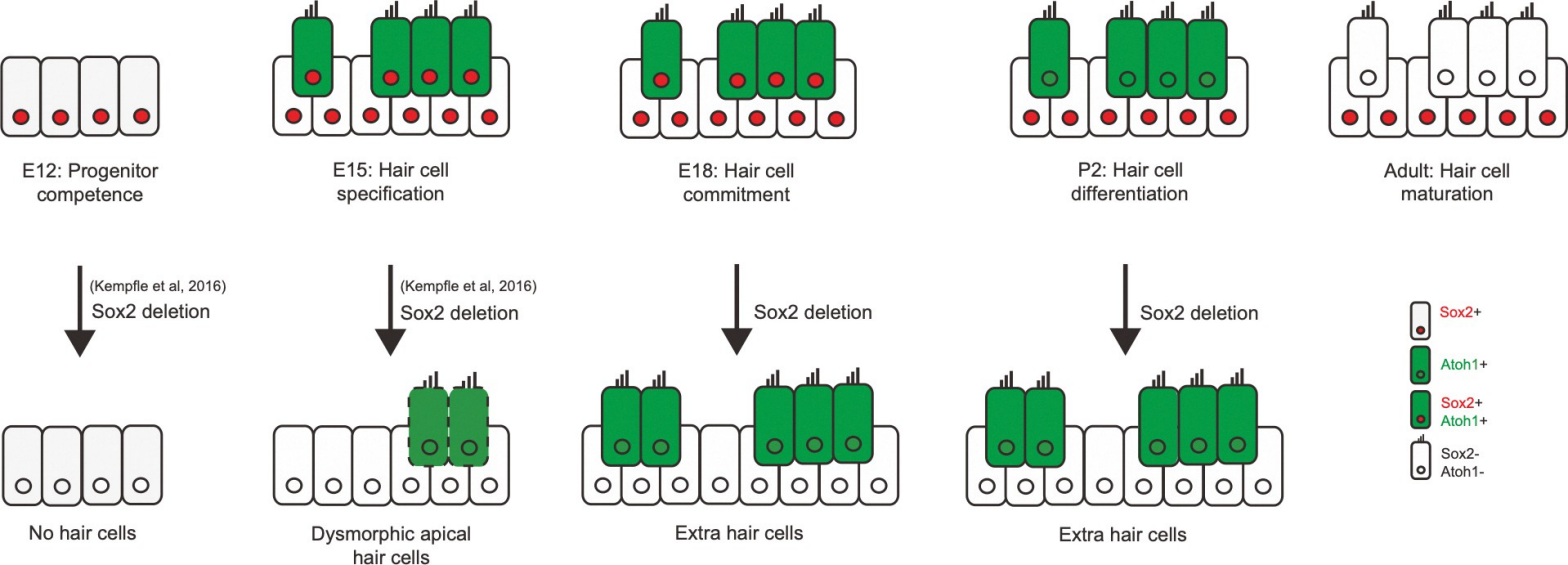
Cochlear phenotypes obtained after *Sox2* deletion. *Sox2* is expressed in cochlear supporting cells and hair cells, both of which are derived from prosensory cochlear progenitors. The time of treatment is plotted from left to right and shows that prosensory cochlear progenitor development requires *Sox2* and that after *Atoh1* expression, *Sox2* switches from its role in keeping proneural genes inactive and allowing the expansion of progenitors to a proneural role by activating transcription of *Atoh1*. At E18, *Sox2* is needed to prevent overexpression of Atoh1 protein through activation of Huwe1, thus exemplifying its role in the initiation of cell fate. *Sox2* is silenced shortly after the establishment of the hair cells at birth.

Co-activation of ubiquitin E3 ligases and transcription factors by Sox2 is potentially a general mechanism for negative feedback control of transcription factor expression as suggested by its binding to 317 E3 ubiquitin ligase genes, as well as over one thousand transcription factor genes [1,11,37,38]. Posttranslational mechanisms are known to impose limits on the effects of chromatin interacting proteins such as transcription factors [53,54]. PEST sequences and other degrons limit the half-life of powerful chromatin-binding proteins such as the intracellular domain of Notch [55], and mutations in these regions can be oncogenic [54]. Countering transcriptional activity by proteasomal degradation is a conserved mechanism that has been demonstrated in plants [56], but has not been observed for a stem cell pluripotency gene like Sox2 that co-initiates expression and degradation. Extra hair cells after *Sox2* conditional knockout at late time points of embryonic development indicated a changing effect of the transcription factor compared to earlier embryonic time points [10] when knockout caused a loss or complete absence of hair cells (Fig 6).

Additional upstream regulators of E3 ligases could contribute to the control of proneural transcription factors [33,57]. Atoh1 protein level in the cerebellum is influenced by *Shh* [33] and *BMP* [33,58]. *Huwe1* is critical for neurogenesis in the cerebellum and acts through both Atoh1 and n-Myc [53,57,59]. Deletion of *Huwe1* in the cerebellum leads to increased production of cerebellar granule cells at the expense of their differentiation. After birth, in the absence of *Shh*, *Huwe1* deletion prevents differentiation due to the high level of Atoh1, which continues to drive proliferation. The loss of *Sox2* led to supporting cell division as reported by others [60]. This appeared to be a result of the lowered activity of *Huwe1* and is likely due to stabilization of targets other than Atoh1.

*Atoh1* overexpression induces new hair cells [61], and triggering the differentiation of new hair cells is a potentially useful approach to the treatment of deafness caused by hair cell loss. However, this effect is limited in the adult cochlea. This is an important limitation of our study which was performed in newborns. Several studies have shown that *Atoh1* transcripts increase in supporting cells after damage [62–64] and recent work has shown regeneration of hair cells in the adult utricle. Our work indicates for the first time that *Sox2*, in addition to binding and stimulating expression of proneural transcription factor genes, acts to limit pro-differentiation activity by stimulating degradation. We have thus described a new role for *Sox2* in the regulation of a proneural transcription factor.

## Methods

### Ethics statement

All mouse experiments were approved by the Massachusetts Eye and Ear Institutional Animal Care and Use Committee.

### Conditional knockout of Sox2 or Huwe1

The *Sox2-CreERT* mice were described previously [25,65]. The *Sox2-flox* mice were obtained from the Jackson Laboratory (Stock number 013093). The *Huwe1-flox* mice were a gift from Dr. Tak Mak (University of Toronto) [66]. Male *Sox2-CreERT* mice were mated with *Sox2-flox* or *Huwe1-flox* female mice; tamoxifen was injected to mothers of the transgenic mice to generate *Sox2 (Sox2-CreERT/+;Sox2*^*fl/+*^) or *Huwe1* (*Sox2-CreERT/+;Huwe1*^*fl/y*^) conditional knockout mice. *Sox2-CreERT* mice were used as heterozygous *Sox2* deleted mice. Littermates without *Cre* were used as controls.

The *Sox2-CreERT* mouse was genotyped with Cre primers: forward, 5’-TGG GCG GCA TGG TGC AAG TT-3’ and reverse, 5’-CGG TGC TAA CCA GCG TTT TC-3’. The *Sox2-flox* mice were genotyped by PCR according to Jackson Laboratory recommendations. The *Huwe1-flox* mice were genotype with the following primers: forward, 5´-GTA TGG TCA TGA TTG AGT GCT TGG AAC T-3´ and reverse, 5´-TAT ACC TGA ACA CAT GGG CAT ATA CAT-3´.

For embryonic cochlea, tamoxifen (250 mg/kg, Sigma-Aldrich, T5648) was given to the pregnant mice (intraperitoneal administration, once a day, for 2 consecutive days) at E17 to obtain conditional knockout of *Sox2*. For postnatal cochlea, tamoxifen (250 mg/kg, Sigma-Aldrich, T5648) was given to the lactating mother (intraperitoneal administration, once a day, for 2 consecutive days) at P0 to obtain conditional knockout of *Sox2* and *Huwe1*, followed by EdU (5-ethynyl-2’-deoxyuridine, 10 mg/ml, Thermo Fisher Scientific, A10044**)** subcutaneous injection at P2 and P3. They were sacrificed at the indicated time points and processed as whole-mount or section preparations.

### Immunohistochemistry

For whole-mount staining of the cochlea, tissue was harvested by removal of the cochlear capsule, lateral wall, and spiral ganglion. The organ of Corti were fixed for 10 minutes in 4% paraformaldehyde, followed by 3 washes with PBS/10% donkey serum (Sigma-Aldrich, D9663)/0.1% Triton X-100 (Sigma-Aldrich, T8787) for 1 hour. Tissues were incubated overnight with PBS/1% BSA/ 0.3% Triton X-100 containing rabbit anti-myosin VIIa antibody (1:500, Proteus BioSciences, 25–6790) and rabbit anti-Sox2 (1:500, Santa Cruz Biotechnology, sc-17320). Samples were washed with PBS for 10 minutes and incubated with secondary antibodies conjugated with Alexa Fluor 488, 594 or 647 (Thermo Fisher Scientific, A-11034, A-21207, A-31573). EdU labeling was performed using the Click-iT EdU kit (Thermo Fisher Scientific, C10339) following the manufacturer’s instructions. Nuclei were visualized with DAPI (4,6-diamidino-2-phenylindole; Vector Laboratories, H-1200).

For sectioning of embryonic cochlea, embryos were harvested at E21 and were fixed in 4% paraformaldehyde for 4 hours at 4° C. After dehydration with sucrose (5% and 30%, Sigma-Aldrich, S7903, S0389), embryos were embedded in OCT (Tissue-Tek, 4583) and kept at -80° C. Tissue was cut (12 μm) and then stained for immunohistochemistry. Fixed tissue sections were dried at room temperature after storage at– 80° C followed by rehydration, washing in PBS, and fixing in 4% paraformaldehyde/PBS for 2x10 minutes. Citric acid buffer was used for antigen retrieval (Abcam), followed by permeabilization and blocking in PBS/15% heat-inactivated donkey serum/0.3% Triton X-100 for 1 hour. Diluted primary antibody PBS/10% heat-inactivated donkey serum/0.1% Triton X-100 was applied overnight at 4° C. Incubation with secondary antibodies was performed for 2 hours at room temperature. Nuclei were visualized with DAPI. Staining was analyzed with epifluorescence (Axioskop2 Mot Axiocam, Zeiss) and confocal (SP8, Leica Microsystems) microscopy.

### Cell culture

HEK 293T cells were grown in DMEM (Thermo Fisher Scientific, 11965092) supplemented with 10% heat-inactivated fetal bovine serum (Thermo Fisher Scientific, 10437028), 2 mM Glutamax (Thermo Fisher Scientific, 35050061) and penicillin (100 U/ml)/streptomycin (100 μg/ml) (Thermo Fisher Scientific, 15140122). *Sox2-HA* (*pCAG-HA-Sox2-IP*, Addgene, No.13459) was transfected into cells at a concentration of 1 μg/ml using Lipofectamine 3000 (Thermo Fisher Scientific, #L3000015) at a ratio of 2 μl Lipofectamine per 1 μg DNA. HEK 293T cells were harvested 24 hours post-transfection.

OC-1 cells (a gift from Dr. Federico Kalinec, University of California, Los Angeles) [67] were cultured under permissive conditions (33° C) in DMEM (Thermo Fisher Scientific, 11965092) supplemented with 10% fetal bovine serum (FBS, Thermo Fisher Scientific, 10437028) and 50 U/ml γ-interferon (Genzyme, 100–09) without antibiotics, and moved to non-permissive conditions (39° C in DMEM supplemented with 10% FBS) prior to qPCR and Western blotting. HEK 293T cells were grown in DMEM (Thermo Fisher Scientific, 11965092) supplemented with 10% heat-inactivated fetal bovine serum, 2 mM Glutamax (Thermo Fisher Scientific, 35050061) and penicillin (100 U/ml)/streptomycin (100 μg/ml) (Thermo Fisher Scientific, 15140122). A tetracycline-inducible *Sox2* embryonic stem cell line was a gift from Dr. Angie Rizzino (University of Nebraska) [35]. It was cultured with 50% DMEM (Thermo Fisher Scientific, 11965092), 50% Ham’s F12 (Corning, 10-080-CV), and penicillin/streptomycin (Thermo Fisher Scientific, 15140122). Doxycylcine hyclate (Sigma-Aldrich, D9891) was added to the cells at 0, 1, 2 4 ng/ml prior to incubation for 48 hours. All cultures were maintained in a 5% CO_2_/20% humidified incubator (Forma Scientific).

*Sox2-HA* (*pCAG-HA-Sox2-IP*) was from Addgene (No. 13459). Plasmids were transfected into cells using Lipofectamine (Thermo Fisher Scientific, 11668019). Sox2 and Atoh1 were cloned into pcDNA 3.1 (Thermo Fisher Scientific, V79020) and transfected into HEK 293T and OC-1 cells; empty vector was used as a control. HEK 293T cells were harvested after 24 hours, and OC-1 cells were harvested after 48 hours.

### Organoid culture

The cochlea from neonatal CD1 mice was harvested at postnatal day 2–3 (P2-P3) and dissected in Hank’s balanced salt solution (HBSS). After treated with Cell Recovery Solution (Corning, 354253) for 1 hour, the cochlear epithelium was peeled from the underlying mesenchyme, collected and treated with Accumax for 20 minutes at 37°C.

To obtain a single cell suspension, cochlear epithelia were mechanically triturated and filtered through a 40 μm cell strainer. The cells were resuspended in Matrigel (Corning, 356231) and seeded in a 24-well plate, using one cochlea per well in average. The cells were cultured in a serum free expansion medium containing a 1:1 mixture of DMEM and F12, supplemented with Glutamax (Thermo Fisher Scientific, 10565–042), N2 supplement (Thermo Fisher Scientific, no.17502-048), B27 supplement (Thermo Fisher Scientific, 17504–044), HEPEs (Thermo Fisher Scientific, no.15630-080), Fungizone Antimycotic (Thermo Fisher Scientific, 15290–018) and Ampicillin sodium salt (Sigma-Aldrich, A0166). The organoids were proliferated with expansion medium added EGF (50 ng/mL; Peprotech, AF-100-15), bFGF (50 ng/mL; Peprotech, 100-18B), IGF-1 (50 ng/mL; Peproech, 291-G1-200), and small molecules CHIR99021 (3 μM; Cayman, 13122), valproic acid (1 mM; Sigma-Aldrich, P4543-10G) and phosphor-vitamin C (280uM; Sigma-Aldrich, 49752-10G) for 10 days. For differentiation, the treatment was replaced by expansion medium with LY411575 (10 μM; Cayman, 16162) and CHIR99021 (3 μM; Cayman, 13122) for two days.

### Gene knockdown

ShRNA used for knockdown of *Huwe1* (TRCN0000073304; hairpin sequence: CCACACTTTCACAGATACTAT) was purchased from Sigma-Aldrich, (SHCLNG-NM_001113396, St. Louis, MO, USA). ShRNA (1 μg/ml) was transfected into cells using Lipofectamine 2000 (Thermo Fisher Scientific, No. 11668019), and transfected cells were harvested 24 hours later. Scrambled shRNA (Sigma-Aldrich, SHC002) was transfected in parallel as a control.

### Cycloheximide chase assay for protein stability

HEK 293T cells were transfected with *FLAG-Atoh1* (1 μg/ml), *Sox2-HA* (1 μg/ml), and *Huwe1* shRNA or scrambled shRNA (1 μg/ml) using Lipofectamine 2000 for 48 hours. Cells were then treated with 100 μg/ml cycloheximide (Sigma-Aldrich, C7698) and harvested at 0, 30, 60, 120, 240 minutes after treatment. Protein lysates were extracted and Western blotted for anti-FLAG (Sigma-Aldrich, F1804) and β-actin (Sigma-Aldrich, A3854). Densitometry of the bands was measured by ImageJ software (NIH). The half-life of FLAG-Atoh1 protein was calculated with a one phase decay model using GraphPad Prism 10 (GraphPad Software, San Diego, CA).

### Western blotting

Western blot analyses were performed as described previously [32]. Proteins extracted with RIPA buffer from whole cells were separated on 4–12% NuPAGE Bis-Tris gels (Thermo Fisher Scientific, NP0321BOX) and electrotransferred to 0.2 μm nitrocellulose or PVDF membranes (BioRad). The membranes were probed with mouse anti-FLAG (Sigma-Aldrich, F1804), mouse anti-HA (Sigma-Aldrich, H3663), anti-Atoh1 (1:1,000, Affinity BioReagent Antibody, OPA1-03050), mouse anti-β-actin (Sigma-Aldrich, A3854), or mouse anti-HSC70 (1:10,000, Santa Cruz Biotechnology, sc-7298) antibodies, followed by HRP-conjugated anti-mouse IgG (Jackson Immunoresearch Laboratories, 115-035-003). The blots were processed with ECL Western Blot Substrates (Thermo Fisher Scientific, 32106). Densitometry by Quantity One software (Bio-Rad, Hercules, CA) was used to quantify the band density. Bands were normalized to β-actin or HSC70 and expressed as a ratio to the control.

### RNA preparation for quantitative RT-PCR

RNA preparation for qRT-PCR analysis was performed as described previously [32]. The qPCR was run in triplicate on a StepOnePlus Real-Time PCR System (Thermo Fisher Scientific, 4376600) with the initial denaturation at 95° C for 2 minutes, denaturation at 95° C for 15 seconds, and annealing/extension at 60° C for 1 minute for 45 cycles. Gene expression was calculated relative to *Gapdh* RNA, and the amount of cDNA applied was adjusted to bring the Ct value for *Gapdh* RNA to within one half-cycle.

### Chromatin immunoprecipitation

The chromatin immunoprecipitation was described previously [22]. In brief, HEK 293T cells were transfected with *Sox2-HA* (1 μg/ml) using Lipofectamine 2000 (2 μl/1 ug DNA, Thermo Fisher Scientific, 11668019) for 24 hours. Cells (2x10^6^ in a 75 cm^2^ culture flask) were cross-linked with 1% formaldehyde for 10 minutes at room temperature, and chromatin was enzymatically sheared into segments of 300–500 bp with Express kit (Active Motif, 53008). The chromatin fragments were immunoprecipitated with 1 μg of mouse anti-HA antibody or nonimmune mouse IgG (both from Sigma-Aldrich). Precipitated DNA was analyzed by quantitative real-time PCR using SYBR Green Realtime PCR Master Mix (Thermo Fisher Scientific, A25742). For each experiment, the percentage of input was determined and normalized to the value obtained at the *Gapdh* gene. All primers are listed in S1 and S2 Tables.

### Chromatin immunoprecipitation and qPCR

Organoids were treated with cell recovery solution for up to 2 hours and TrypleE Express Enzyme (Thermo Fisher Scientific, 12604013) for 30 minutes. More than 6 wells organoids or about 2x10^5^ HEK 293T cells (10 cm^2^ culture plate) were collected and cross-linked with 1% formaldehyde for 10 minutes at room temperature before termination with 0.125M Glycine. The cells were treated with lysis buffer (0.5 M EDTA and 0.05% Triton-X100) for 30 minutes on ice and then with nuclear extraction buffer (0.5 M EDTA, 20% SDS, 1 M Tris-HCl, pH 8) for 10 minutes. The cross-linked chromatin was sonicated for 10 minutes (HEK 293T cells) or 30 minutes (LCPs) (30x30 seconds with 30 seconds intervals), and the shearing quality (sheared into segments of 200–1000 bp) was confirmed by running 10 μl of the sample on a 1% agarose gel. Lo-bind tubes containing Dyna-A and Dyna-G beads (Thermo Fisher Scientific, 10001D, 10003D) in PBS-BSA were incubated with 3 μg of primary antibody for 6 hours-overnight with rotation at 4° C. The antibodies used were as follows: anti Sox2 antibody (R&D System, AF2018), anti HA antibody (Sigma-Aldrich, H3663), goat IgG (Sigma-Aldrich, I9140), normal mouse IgG (Sigma-Aldrich, 12–371). The chromatin samples were treated with dilution buffer (1% Triton-X 100, 0.5 M EDTA, 5 M NaCl, 1 M Tris-HCl, pH 8 and 1% protease inhibitors x 100) and incubated with the prepared beads for 6 hours-overnight. The beads were captured on a magnetic rack, washed with low salt wash buffer (0.1% SDS, 1% Triton X-100, 2mM EDTA, 20 mM Tris-HCl, 150 mM NaCl), high salt wash buffer (0.1% SDS, 1% Triton X-100, 2 mM EDTA, 20 mM Tris-HCl, 500 mM NaCl), LiCl wash buffer (0.25 M LiCl, 1% NP 40, 1% sodium deoxycholate, 1mM EDTA, 10 mM Tris-HCl) twice each for 10 minutes, and rinsed with TE buffer (10 mM Tris-HCl and 1 mM EDTA). The beads were resuspended in elution buffer (0.1 M NaHCO3 and 1% SDS) and incubated at 65° C for 30 minutes. The beads were collected by the magnetic rack, and the supernatant were transferred to reverse cross-links by adding 5 M NaCl followed by incubating 6 hours-overnight at 65° C. The DNA was purified using phenol:chloroform extraction.

Precipitated DNA was analyzed by quantitative real-time PCR using SYBR Green Realtime PCR Master Mix (Thermo Fisher Scientific, A25742) with specific primers (S1 and S2 Tables). The results were calculated as relative fold enrichment according to negative IgG controls.

### Statistical analysis

Data were analyzed for significance by an unpaired two-tailed Student’s t-test or ANOVA with indicated alpha (0.05, 0.01 or 0.001) by GraphPad Prism 10 software.

All values plotted in the figures and all Supporting Information have been deposited in Dryad [68]

### Dryad DOI

http://datadryad.org/stash/share/NDFufE0XhDncepb0MfFkyL2AB_9qn3LHwOUzEvUw9jo

## Supporting information

S1 Fig Increased *Huwe1* after induction of Sox2 in mouse ES cells. The expression of *Sox2* increased after doxycycline (Dox) induction in a transgenic embryonic stem cell line based on quantitative RT-PCR. Elevated expression of *Huwe1* correlated with the upregulation of *Sox2*. *Atoh1* expression increased with *Sox2* at low levels but decreased at higher *Sox2* level. The relative expression of each gene increased significantly (*p < 0.05) compared to untreated controls (no Dox). Error bars indicate SEM (n = 4 independent experiments).
(TIF)

S1 Table Primer pairs for Sox2 ChIP upstream and downstream of the mouse *Huwe1* gene. (DOCX)

S2 Table Primer pairs for Sox2 ChIP upstream and downstream of the human *Huwe1* gene. (DOCX)

S3 Table Source of antibodies. (DOCX)

S4 Table Identification of Sox2 target transcription factor and ubiquitin E3 ligase genes. To identify Sox2 targets that are transcription factors and ubiquitin E3 ligases, we used databases of human transcription factors and ubiquitin E3 ligases. The supplementary table lists the intersections between Sox2 transcriptional targets and transcription factors (column 1), and ubiquitin E3 ligases (column 2). Sox2 transcriptional targets are integrated from four independent databases (http://tfbsdb.systemsbiology.net/; https://doi.org/10.1016/j.gpb.2019.09.006; https://doi.org/10.1093/nar/gkx1013;https://maayanlab.cloud/Harmonizome/; http://bioinfo.life.hust.edu.cn/HumanTFDB/#!/; https://esbl.nhlbi.nih.gov/Databases/KSBP2/Targets/Lists/E3-ligases/).
(XLSX)

S5 Table Identification of ubiquitin E3 ligases and substrates bound by Sox2. The supplementary table lists the ubiquitin E3 ligases and substrates targeted by Sox2. Substrates are highlighted in blue and E3 ligases are in yellow. Sox2-targeted substrates and the associated E3 ligases were identified using the UbiBrowser database (http://ubibrowser.bio-it.cn/ubibrowser_v3/).
(XLSX)
