# Supplementary figures and images for: Sox2 interacts with Atoh1 and Huwe1 loci to regulate Atoh1 transcription and stability during hair cell differentiation
Source: PLoS Genet. 2025 Jan 30;21(1):e1011573. doi: 10.1371/journal.pgen.1011573 (PMC11813075; doi:10.1371/journal.pgen.1011573)

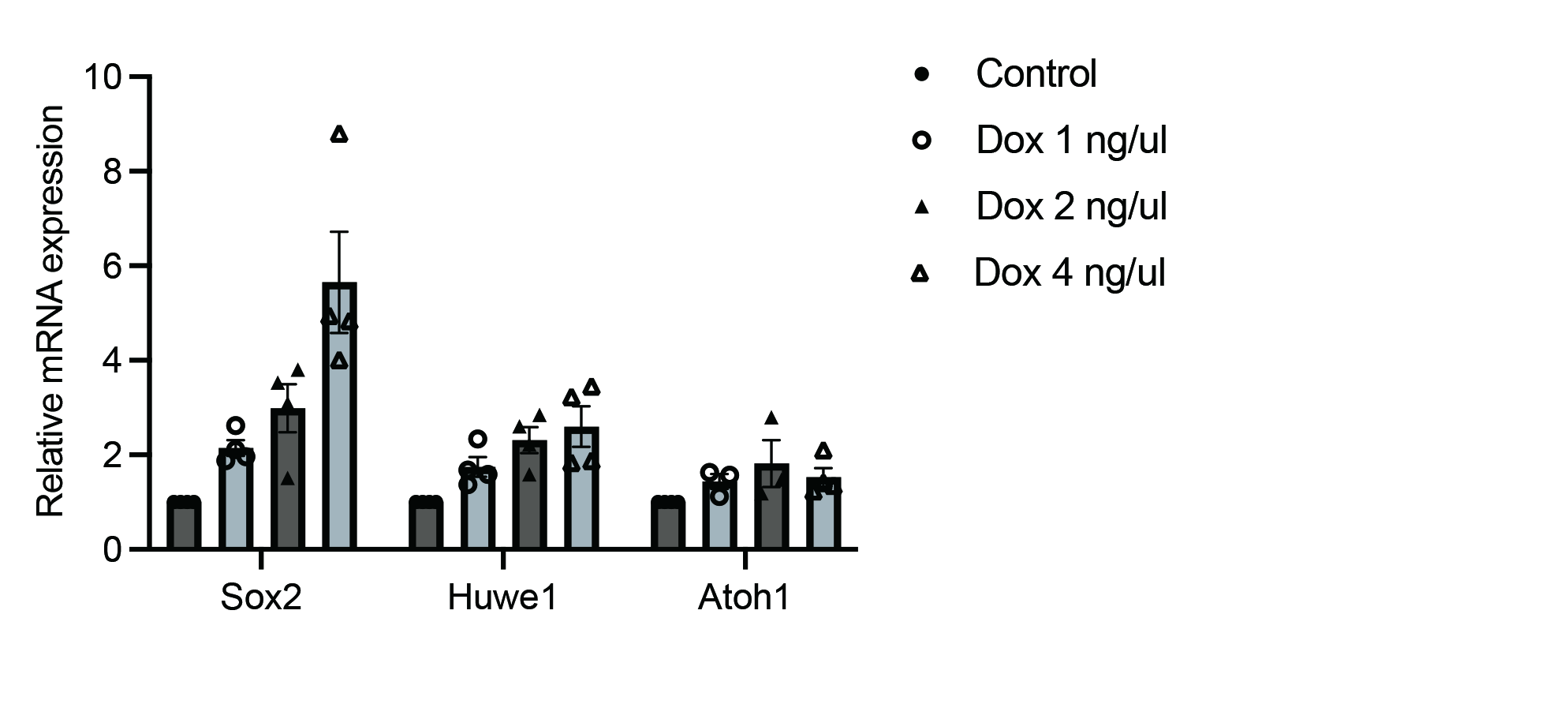

Supplement: S1 Fig — The expression of Sox2 increased after doxycycline (Dox) induction in a transgenic embryonic stem cell line based on quantitative RT-PCR. Elevated expression of Huwe1 correlated with the upregulation of Sox2. Atoh1 expression increased with Sox2 at low levels but decreased at higher Sox2 level. The relative expression of each gene increased significantly (*p < 0.05) compared to untreated controls (no Dox). Error bars indicate SEM (n = 4 independent experiments). (TIF) [file pgen.1011573.s001.tif]
