# Supplementary material for: Sox2 interacts with Atoh1 and Huwe1 loci to regulate Atoh1 transcription and stability during hair cell differentiation
Source: PLoS Genet. 2025 Jan 30;21(1):e1011573. doi: 10.1371/journal.pgen.1011573 (PMC11813075; doi:10.1371/journal.pgen.1011573)
Supplement: S1 Table — (DOCX) [file pgen.1011573.s002.docx]

**S1 Table**

**Primer pairs for Sox2 ChIP upstream and downstream of the mouse *Huwe1* gene**

| **Putative motif** | **Position relative to translation start site*** | | **Forward primer** | **Reverse primer** |
| --- | --- | --- | --- | --- |
| **1** | -79798/-79583 | 5’- GCCCTCTAATCTTCCTCCACA -3’ | | 5’- AGTACCATGTCTGCCTGAGTT -3’ |
| **2** | -74018/-73718 | 5’- CAGATACAGTGCCAGGATGT -3’ | | 5’- TCTCAGCAGCCTATAGAACTTTC -3’ |
| **3** | -32743/-32553 | 5’- CCTTTCTCCCCACCCCAAG -3’ | | 5’- CCGCACTGCAGGCTAACC -3’ |
| **4** | -6237/-6049 | 5’- AGTGCTAGCTTGGTTGTTATTGA -3’ | | 5’- CAGACATAGCCCAGACTTTTCA -3’ |
| **Putative motif** | **Position relative to stop codon**** | **Forward primer** | | **Reverse primer** |
| **5** | -54/+171 | 5’- CCACATGCTACTATTGGCCA -3’ | | 5’- GAAGCGAACAGGTATGCACT -3’ |
| **6** | +6655/+6832 | 5’- CTCCCGAGTGCCAGGATATA -3’ | | 5’- GCCCTTATCTGAAGAGTCTGC -3’ |
| **7** | +40629/+40838 | 5’- CTAACTCACCCAGCGGCC -3’ | | 5’- ACATCATACCAGAGACAGGCT -3’ |
| **8** | +14420/+14636 | 5’- CAATGTGATGGCTTGCTCCC -3’ | | 5’- TCTGAATCCAAAGGCACCTG -3’ |

** Positions are 5’ relative to the translation start site of mouse Huwe1.*

*** Positions are 3’ relative to the stop codon of mouse Huwe1.*
