# Supplementary material for: Sox2 interacts with Atoh1 and Huwe1 loci to regulate Atoh1 transcription and stability during hair cell differentiation
Source: PLoS Genet. 2025 Jan 30;21(1):e1011573. doi: 10.1371/journal.pgen.1011573 (PMC11813075; doi:10.1371/journal.pgen.1011573)
Supplement: S2 Table — (DOCX) [file pgen.1011573.s003.docx]

**S2 Table**

**Primer pairs for Sox2 ChIP upstream and downstream of the human *Huwe1* gene**

| **Putative motif** | **Position relative to translation start site*** | | **Forward primer** | **Reverse primer** |
| --- | --- | --- | --- | --- |
| **A** | -42018/-41828 | 5’-ACAGCCAAGTGTTGCTAGGG-3’ | | 5’-GCTCCCTTTGTGTCTGCTTC-3’ |
| **B** | -34389/-34178 | 5’-TAAGCCCTAATCGCACCAGT-3’ | | 5’-TCTTTGACTATTACAGTGGGGTTTT-3’ |
| **Putative motif** | **Position relative to stop codon**** | **Forward primer** | | **Reverse primer** |
| **C** | +2840/+3049 | 5’-AGCGAGACCCCGTCTAAAAA-3’ | | 5’-CGCCCAGCCTGAAACTTTAT-3’ |
| **D** | +3615/+3818 | 5’-AGGCTGGTCTTCAACTCCTG-3’ | | 5’-TGACGGTAGAGGTTGCAGTG-3’ |
| **E** | +7695/+7905 | 5’-GGTCCCTCCCTCGACATAGT-3’ | | 5’-ACCCCTCCTCTTCCTCTTCA-3’ |
| **F** | +6071/+6262 | 5’-GTTCTCCATGAGGGCTTCAC-3’ | | 5’-GCAAAAGTTGCAGTGAGCTG-3’ |

** Positions are 5’ relative to the translation start site of human Huwe1.*

*** Positions are 3’ relative to the stop codon of human Huwe1.*
