# Supplementary material for: Sox2 interacts with Atoh1 and Huwe1 loci to regulate Atoh1 transcription and stability during hair cell differentiation
Source: PLoS Genet. 2025 Jan 30;21(1):e1011573. doi: 10.1371/journal.pgen.1011573 (PMC11813075; doi:10.1371/journal.pgen.1011573)
Supplement: S3 Table — (DOCX) [file pgen.1011573.s004.docx]

**S3 Table**

**Source of antibodies**

| **Antibody** | **Company** | **Cat #** |
| --- | --- | --- |
| Sox2 | R&D | AF2018 |
| HA | Sigma | H3663 |
| Goat IgG | Sigma | I9140 |
| Normal mouse IgG | Sigma | 12-371 |
